# Cholinergic substrates of gait and postural impairments in Progressive Supranuclear Palsy

**DOI:** 10.21203/rs.3.rs-8778777/v1

**Published:** 2026-02-04

**Authors:** Prabesh Kanel, Giulia Carli, Stiven Roytman, Sygrid van der Zee, Robert Vangel, Jaimie Barr, Taylor Brown, C Chauncey Spears, Amanda Narkis, Sofie Slingerland, Sanne Meles, Teus van Laar, Peter J.H. Scott, Roger L. Albin, Nicolaas I. Bohnen

**Affiliations:** University of Michigan; University of Michigan

**Keywords:** Progressive Supranuclear Palsy, cholinergic deficits, PET imaging, PIGD

## Abstract

**Purpose:**

Progressive Supranuclear Palsy (PSP) is an atypical parkinsonian syndrome characterized by significant postural instability and gait difficulties (PIGD). While brain cholinergic deficits are documented in PSP, their role in the pathophysiology of PIGD is an area of active research. This cross-sectional study aimed to elucidate relationships between regional cholinergic denervation, assessed by [^18^F]FEOBV PET, and PIGD severity in PSP patients.

**Methods:**

Nineteen subjects characterized clinically as PSP (twelve males, seven females; mean age of 69.47 ± 6.46 years [range 55–79]). Based on the Movement Disorders Society-PSP diagnostic criteria, sixteen patients had probable PSP (eleven males; five females) and three had suggestive PSP (one male; two females). Clinical assessments showed significant motor impairments, a mean MDS-UPDRS Part III “off state” score of 42.36 ± 13.52 and a mean modified Hoehn and Yahr stage of 3.36 ± 1.22.

**Results:**

Statistical parametric mapping (SPM) based voxel-wise analysis of [^18^F]FEOBV PET data revealed a significant inverse correlation between lower regional [^18^F]FEOBV binding and more severe PIGD motor rating scores. This association was observed across brain regions, including orbitofrontal cortices, gyrus rectus, septal nuclei, medial temporal lobe, insula, metathalamus, dorsomedial thalamus, pericentral cortices, caudate nuclei, anterior greater than mid and posterior and retrosplenial cingulate cortices, frontal lobe, and cerebellum.

**Conclusions:**

These findings highlight the potential roles of cholinergic systems degenerations in mediating PIGD in PSP. This suggests that cholinergic systems degeneration plays a substantial role in the pathophysiology of PIGD in PSP, offering a potential avenue for targeted therapeutic interventions to improve mobility and quality of life for these patients.

## Introduction

Progressive supranuclear palsy (PSP) is a debilitating neurodegenerative disorder characterized by early-onset and severe postural instability and gait difficulties (PIGD), often leading to frequent falls [1, 2]. While PSP shares some clinical features with Parkinson disease (PD), particularly the presence of balance and gait impairments, PSP’s underlying neuropathology and neurochemical changes are significantly different. Unlike the α-synucleinopathy of PD, PSP is primarily associated with the accumulation of 4-repeat (4R) tau protein deposits in neurons and glial cells, particularly in subcortical regions, along with characteristic atrophy of the subthalamic nuclei, midbrain, and superior cerebellar peduncles [3–6].

In contrast to PD, where falls tend to occur later in disease progression, PSP patients experience falls in early disease, often falling backwards due to postural rigidity [7]. Early onset and severity of PIGD in PSP poses a significant clinical challenge, as falls are a leading cause of morbidity and mortality in this population [8, 9]. While loss of nigrostriatal dopaminergic function is a major contributor to motor impairments in PD [10, 11], its role in PSP features is less clear. Studies show dopaminergic losses in PSP [12, 13], but responses to dopamine replacement therapies are generally limited. Poor responses to dopaminergic replacement suggest that apart from post-synaptic dopaminergic system pathologies, other neurotransmitter systems are involved in the pathophysiology of PIGD in PSP. In PD, cholinergic deficits involving the (meta-) thalamus, striatum, hippocampus, amygdala, and some cortical regions were shown to be associated with episodic PIGD motor features (falls and freezing of gait) [10, 14]. These results suggested a cholinergic deficits-based systems-level model of PIGD pathophysiology in PD, which might generalize to PSP. Although the regional distribution of PIGD-related cholinergic system deficits is characterized in PD, there is lack of data regarding cholinergic changes associated with PIGD in PSP.

Our previous research, using vesicular acetylcholine transporter (VAChT) [^18^F]Fluoroethoxybenzovesamicol ([^18^F]FEOBV) PET imaging, revealed widespread cholinergic deficits in PSP, more severe and extensive than those observed in PD patients [15]. The affected areas included the tectum, metathalamus, epithalamus, pulvinar, bilateral frontal opercula, anterior insulae, superior temporal pole, anterior cingulate, several striatal subregions, the lower brainstem, and the cerebellum. This result suggests that cholinergic systems are involved more extensively as the substrates of PIGD in PSP.

This study aims to investigate *in vivo* regional cortical and subcortical cholinergic denervation in PSP patients as related to PIGD using VAChT [^18^F]FEOBV PET imaging. We hypothesize that cholinergic system changes play a role in the PIGD motor features in PSP and may be due to extensive subcortical (striatal, brainstem, thalamic and cerebellar) losses. By elucidating the regionally specific cholinergic deficits associated with PIGD in PSP, this study aims to inform the development of novel therapeutic strategies targeting cholinergic dysfunction in PSP.

## Material and Methods

### Study design and participants

Sixteen PSP subjects were recruited from the Atypical Parkinsonism Clinic at Michigan Medicine. Three subjects were part of the Dutch Parkinson Cohort (DUPARC) study at the University of Groningen Medical Center in the Netherlands [16]. Participant recruitment and assessment protocols varied between sites. In Groningen, individuals later identified with PSP were initially enrolled as having *de novo* PD, and their motor function at the time of imaging was assessed with the Movement Disorder Society Revised Unified PD Rating Scale (MDS-UPDRS). The Michigan study, primarily focused on PD, also incorporated sub-studies designed for PSP. All Michigan participants, including those with PSP, were evaluated with the MDS-UPDRS, with a limited number of PSP patients receiving the PSP rating scale (see Table 1 for available PSP rating scale scores). Subjects with evidence of large vessel strokes or other intracranial lesions on MRI were excluded from the study. The Institutional Review Board of the University of Michigan School of Medicine, and Medical Ethical Committees of the University of Groningen and Veterans Affairs Ann Arbor Healthcare System approved this study (ClinicalTrials.gov Identifier: NCT02458430 & NCT01754168) in compliance with Declaration of Helsinki guidelines. Written informed consent was obtained from all participants (or legal representatives) prior to any study procedures. A subset of subjects from the Michigan site were previously included in an investigation of distinct topographies of cholinergic deficits in PSP compared to PD and neurologically healthy older individuals [15].

Subject classification utilized the 2017 Movement Disorder Society clinical diagnosis criteria for PSP (MDS-PSP) [17]. This framework allows subtyping of PSP based on predominant clinical features and levels of diagnostic certainty. In Michigan, two movement disorder specialists, with expertise in atypical parkinsonian syndromes, retrospectively applied the MDS-PSP criteria. In Groningen, a single movement disorder specialist applied the MDS-PSP criteria along with [^18^F]Fluorodeoxyglucose-PET ([^18^F]FDG PET) imaging to validate the PSP diagnosis at subsequent visits. These assessments focused on clinical features present at the time of imaging. To calculate MDS-UPDRS PIGD sub-scores, we used the sum of items 2.12 (Walking and balance), 2.13 (Freezing), 3.10 (Gait), 3.11 (Freezing of Gait), 3.12 (Postural Stability), 3.13 (Body stooping) from the MDS-UPDRS (Part II and Part III) and self-reported history of falls within the last year (yes or no) [10].

### Imaging acquisition and pre-processing

MRI was performed on a 3 Tesla Philips Achieva system (Philips, Best, The Netherlands) at Michigan Medicine and a 3 Tesla Philips Intera system (Philips, The Netherlands) at the University Medical Center Groningen (UMCG) as previously described [10, 18]. PET imaging was performed using a Biograph 6 TruPoint PET/CT scanner (Siemens Molecular Imaging, Inc., Knoxville, TN) at the university of Michigan and Biograph 40-mCT or 64-mCT TruPoint PET/CT scanner (Siemens Molecular Imaging, Inc., Knoxville, TN) at the UMCG as previously described [18, 19]. [^18^F]FEOBV delayed dynamic imaging was performed over 30 minutes (in six 5-minute frames) starting at 3 hours in Michigan and 3.5 hours in Groningen after intravenous bolus dose injections of 8 mCi [^18^F]FEOBV [18]. To ensure consistent data across PET images from both centers, we implemented a thorough harmonization process for the NC and PD populations to validate our method. This process was later applied to our PSP population. Even though both sites used the same scanner model, we calculated SUVs for the reference region, which reflect actual biological differences rather than site-specific technical variations. We addressed potential discrepancies arising from different acquisition protocols by using the same reconstruction algorithms and standardizing these parameters via post-reconstruction filtering. This approach minimized inter-center bias and established a unified dataset that is suitable for robust statistical analysis. A detail description of data harmonization process across centers available in the reference paper [18].

Equilibrium distribution volume ratio (DVR) parametric PET images were obtained using a reference region normalization approach, with eroded supraventricular cerebral white matter as the reference region. Frames 2–6 of the delayed dynamic [^18^F]FEOBV PET image were rigidly co-registered to frame 1 to correct for motion artifacts. The dynamic PET images were subsequently averaged across frames. To obtain the reference region mask, structural MR images were AC-PC-aligned and segmented using the standard FreeSurfer software suite (https://surfer.nmr.mgh.harvard.edu/). Cerebral white matter labels were truncated below the lateral ventricle and eroded with a 3mm radius sphere in MRI space. A rigid-body transformation from MR to PET space was computed and applied to the reference region labels, which were then resampled to PET resolution using nearest neighbor interpolation. Finally, parametric images were generated in PET space by dividing voxel values in each frame-averaged dynamic PET image by the mean activity of all the voxels belonging to the reference region.

Structural MR images went through high-dimensional DARTEL registration and spatial normalization into Montreal Neurological Institute (MNI) space template. Using the information from MR, the registered parametric PET images were transformed to MNI space as previously described [15]. Correction for partial volume effects before standardizing into MNI space was done using Müller-Gartner method [20]. A smoothing of 8 mm full width at half maximum (FWHM = 8mm) was applied to remove random noise.

### [^18^F]FEOBV Imaging analyses

#### Voxel-wise approach:

To explore regional cholinergic deficit correlates of PIGD features in PSP without any predefined restrictions (no a priori hypotheses), we conducted a whole-brain voxel-wise regression analysis using SPM12. PIGD scores were used as the independent variable and [^18^F]FEOBV images as the dependent variable, with sex, disease duration and levodopa equivalent dose (LEDD) as nuisance covariates. Resulting statistical parametric maps were voxel-wise thresholded at an uncorrected p-value cutoff of less than 0.001, and then cluster-level false discovery rate (FDR) multiple comparison correction applied. Clusters with an FDR-corrected q-value of less than 0.01 were considered statistically significant. Primary analysis was performed on images corrected for partial volume effect, but an additional supplementary analysis was also done on images without partial volume correction (PVC; Supplementary Materials Section 1). An additional analysis was conducted using total MDS-UPDRS Part III scores to see the effects of motor symptom severity associated with cholinergic deficits (Supplementary Materials Section 2).

#### Univariate correlation post-hoc:

To obtain the proportion of variance in PIGD scores explained by [^18^F]FEOBV uptake in relevant regions, a post-hoc univariate correlation analysis was performed, using mean [^18^F]FEOBV uptake of the clusters which survived FDR-correction in the voxel-wise analysis as the independent variable and the MDS-PIGD score as the dependent variable. The relationship between the two variables was visualized as a scatterplot with the regression line of best fit. An additional post-hoc sensitivity analysis was performed, examining whether individual subregions contained within the cluster of statistically significant voxels (separated based on the sources of cholinergic innervation) exhibited differential strengths of association with PIGD scores (Supplementary Materials Section 3). An additional supplementary analysis was performed, examining the association between age, center (University of Michigan vs. University of Groningen), and PIGD scores and standardized uptake value (SUV) of the reference region, to support that our findings were not driven by reference region related bias (see Supplementary Materials Section 4 for detailed methods).

## Results

This cross-sectional study included nineteen subjects with PSP, twelve males and seven females, with a mean age of 69.47 ± 6.46 years (range 55–79). Based on the 2017 MDS-PSP diagnostic criteria, sixteen subjects had probable PSP (eleven males; five females) and three had suspected PSP (one males; two females). Subtype analysis revealed thirteen with probable Richardson Syndrome (PSP-RS), one probable PSP with predominant Parkinsonian criteria (PSP-P), and three suggestive PSP with the PSP-P phenotype. Two subjects diagnosed with probable PSP were missing information required for subtyping based on the MDS-PSP criteria and were therefore classified as uncertain subtypes. The mean MDS-UPDRS Part III score in the medication ‘off’ state was 42.36 ± 13.52 and the mean modified Hoehn and Yahr stage was 3.36 ± 1.22. Phenotypic details of study subjects are provided in Table 1. Detailed clinical and demographic characteristics are provided in Table 2.

### Voxel-wise results -

**Figure 1:**
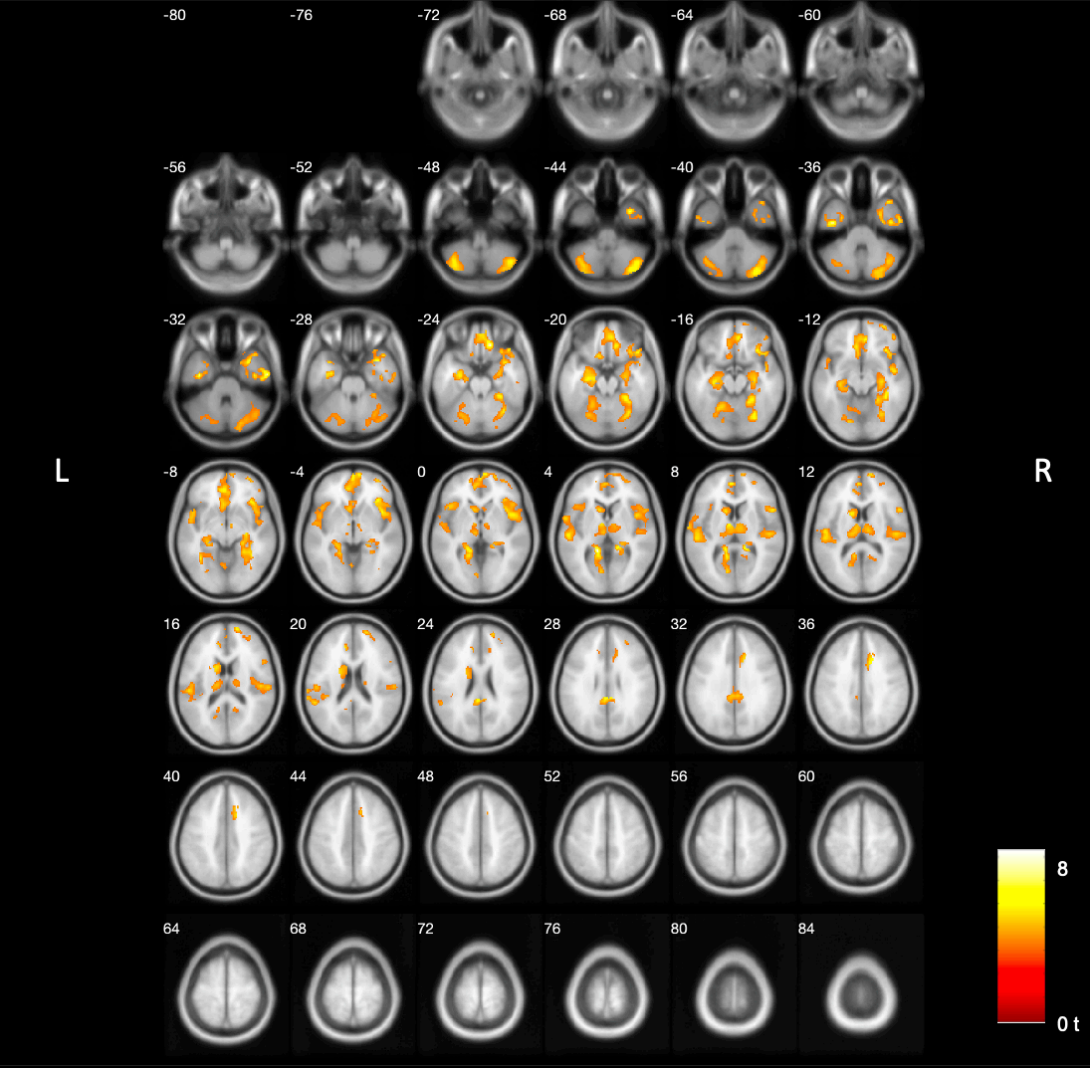
Voxels where [^18^F]FEOBV uptake exhibited statistically significant negative correlation with MDS-PIGD scores in PSP subjects. Color indicates SPM voxel-based regression analysis strength of correlation between lower cholinergic binding and MDS-PIGD scores in PSP subjects with yellower color indicating more robust significant correlations (voxel-level uncorrected p<0.001, cluster-level FDR-corrected at P<0.01; adjusted for sex, disease duration and Levodopa Equivalent Dose).

Voxel-based analysis revealed that lower [^18^F]FEOBV binding in several brain regions (Fig. 1) correlated with more severe PIGD motor rating scores. These associations were observed in the orbitofrontal cortices, gyrus rectus, septal nuclei, medial temporal lobe, insulae, metathalamus, dorsomedial thalamus, pericentral cortices, caudate nuclei, anterior more than the mid and posterior and retrosplenial cingulate cortices, frontal lobe, and cerebellum. Table 3 details the main significant clusters, peak MNI coordinates, and associated regions. Results of analysis on images without PVC applied are presented in Supplementary Materials Section 1. Non-PVC analysis recapitulates the topography of correlated deficits found with PVC corrected images but with more extensive statistically significant voxels observed in the brainstem, pons, and midbrain (Supplementary **Figure S1**). We supplemented our primary analysis with MDS-UPDRS Part III total scores (Supplementary Materials Section 2) to observe cholinergic system deficits associated with overall motor severity. Although both models showed comparable cortical clusters, the PIGD-associated findings uniquely highlighted both cortical (insulae) and subcortical regions (striatum, thalamus, and cerebellum). This suggests that these subcortical associations are specific to PIGD rather than a reflection of global motor impairment (Supplementary Figure S2).

### Univariate post-hoc results –

**Figure 2:**
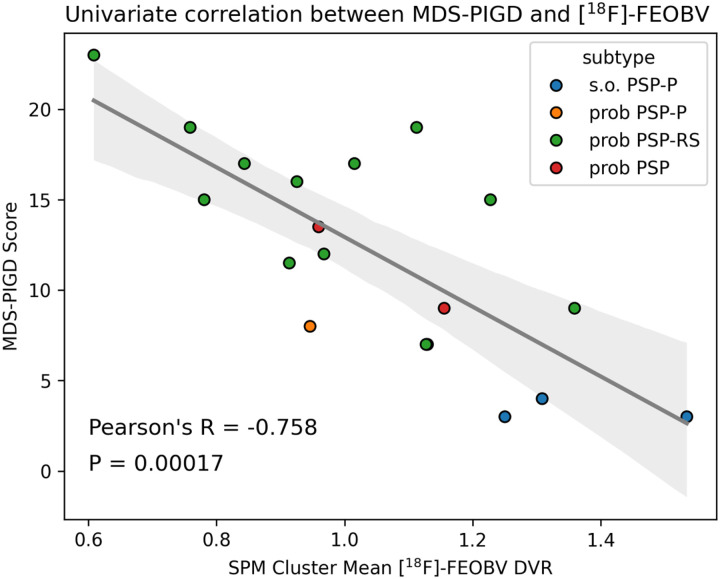
Scatterplot of the univariate correlation between MDS-PIGD scores and mean [^18^F]FEOBV uptake in SPM clusters which survived correction for multiple comparisons. Individual observations are colored based on PSP subtype.

Univariate post-hoc correlation analysis demonstrated that the [^18^F]FEOBV topography from the primary voxel-wise analysis accounts for just over half of the variance in MDS-UPDRS PIGD sub-scores among PSP participants (F = 22.94, *R*^2^ = 0.574, p = 0.00017), with lower [^18^F]FEOBV uptake in the discovered topography correlating with higher MDS-UPDRS PIGD sub-scores (R=−0.758 [−1.092, −0.424]). A scatterplot of the univariate relationship is presented in Fig. 2. The sensitivity analysis demonstrated that the greatest amount of variance in PIGD scores was accounted for by voxels in the hippocampus innervated by medial septum and vertical limb of the diagonal band (*R*^2^ = 0.459) and voxels in the thalamus innervated by the pedunculopontine nucleus (*R*^2^ = 0.451). In either instance, however, variance accounted for did not exceed that obtained from the mean [^18^F]FEOBV uptake of the entire cluster’s assemblage. Supplementary analysis demonstrated that neither age, site, nor PIGD scores associated significantly with reference region [^18^F]FEOBV SUV (Supplementary Materials Section 4). These findings were in agreement with a previously published analysis of inter-site harmonization performed in a larger sample of normal controls and Parkinson’s disease patients [18].

## Discussion

This study employed [^18^F]FEOBV VAChT brain PET imaging to quantify the extent of regional cholinergic denervation in relationship to MDS-UPDRS PIGD sub-scores in subjects with PSP. The results revealed significantly associated regional cholinergic deficits across an assemblage of brain regions. These regions include the anterior cingulate, (meta-)thalamus, striatum, limbic and paralimbic structures (hippocampus, parahippocampal gyrus and the insulae), peri-central cortices and the cerebellum. These results are highly consistent with the topography of cholinergic deficits we previously described in PSP patients compared to controls [15]. The association of cholinergic deficits in these regions with PIGD aligns with previous research describing cortical and subcortical dysfunctions in PSP motor deficits [21–24]. The affected regions are associated with a diverse range of functions, including oculomotor control, sensory integration, and cognitive processing, all of which are likely to contribute to maintenance of posture and gait control [7, 21, 25, 26]. This result suggests that the pathophysiology of PIGD features is likely multifactorial in PSP, potentially arising from multilayered failures in sensory processing, balance, cognition, and motor control. Our results also align with the hypothesis that PSP and its clinical manifestations should be conceptualized as an assemblage of network-based disorders [27]. Our post-hoc sensitivity analysis findings supports this conclusion, given that none of the individual cholinergic innervation target subregions (separated by source of innervation) were as predictive of PIGD severity as the composite of all relevant targets.

Our results implicate degeneration of all major brain cholinergic projections in the pathophysiology of PIGD in PSP. In this PSP cohort, cholinergic deficits in subcortical structures, including the bilateral metathalamus and left more than right caudate nucleus, demonstrated strong associations with PIGD features severity, substantially overlapping with our previous findings in a PD cohort [10], but with additional greater involvement of the cerebellar hemispheres. These regional cholinergic terminal deficits align with the known anatomy of cholinergic nuclei and their projections. The brainstem pedunculopontine tegmental nucleus (PPN) and the laterodorsal pontine tegmentum (LDT) complex are the primary sources of cholinergic projections to the thalamus [28, 29]. The PPN and LDT are implicated in the coordination of gait [30]. Thalamic dysfunction is closely associated with the characteristic gait disturbances and early falls observed in PSP, with dysfunction in the mesencephalic brainstem-thalamic loop playing a crucial role in multisensory postural control [21]. The striatum is characterized by the presence of cholinergic interneurons [28], which play a central role in the basal ganglia circuitry, influencing both the control of voluntary movements and the pathophysiology of movement disorders [31–33]. Cerebellar cholinergic deficits likely reflect degeneration of projections from the medial vestibular complex, another structure with a critical role in postural and gait control.

We also found novel evidence that PIGD motor features in PSP associated with losses of cholinergic forebrain limbocortical and paralimbic projections. These structures significantly influence cognition and behavior, particularly through the frontal-subcortical circuits [34–36]. Such cholinergic deficits suggest a disruption in the complex interplay of these regions and the disruption of broader neural networks subserving mobility. [37–41]. Entorhinal cortex, parahippocampal gyrus and retrosplenial cortex are crucial brain regions for spatial navigation [42–45]. Entorhinal cortex receives vestibular inputs, relayed via thalamic nuclei, providing information about balance and spatial orientation. Entorhinal dysfunction would plausibly lead to problems with spatial memory and navigation [42–44]. Cholinergic forebrain limbic and paralimbic projections and related deficits in attentional function and spatial navigation may be critical contributors to the multifactorial PIGD symptoms in PSP.

Similarly, Anterior Cingulate Cortex (ACC), which connects the supplementary eye field, frontal eye field, and midbrain regions, plays a role in cognitive control and action valuation, including indirect role to those related to saccadic eye movement [25]. Cholinergic deficits in regions suggest a potential link between oculomotor dysfunction and overall motor impairment in PSP. The images without partial volume correction did show correlations with upper brainstem regions that are directly involved with vertical gaze functions. It is possible that the small size of the structures was the reason that findings did not survive partial volume correction. Further research with more detailed brainstem atlases and more sensitive PET images is needed. It is clear that oculomotor impairments with vertical movement difficulty and convergence as well as reduced blinking and involuntary eyelid closure, can influence gait instability, altered step length, and an increase frequency of falls in PSP [46–48]. The ACC participates in both medial frontal-subcortical and dorsolateral prefrontal-subcortical circuits and cholinergic ACC deficits might contribute to apathy and executive dysfunction in PSP [34, 49]. Cognitive problems, such as executive dysfunction, slowed processing speed, attention deficits, and diminished working memory, likely also contribute to the gait instability in PSP [50].

This study has several limitations. The relatively small sample size of 19 PSP subjects, with varying subtypes, may limit the generalizability of the findings. Specifically, the observed patterns of cholinergic denervation may be primarily driven by the PSP-RS subtype, which comprised the majority of the participants. Consequently, we may have overlooked subtle topographic patterns of cholinergic loss that could explain the characteristic features of other PSP subtypes, such as PSP-P. Future studies with larger cohorts, specifically designed to include a balanced representation of different PSP subtypes, are needed to validate these findings and explore potential subtype-specific patterns of cholinergic dysfunction. However, this study provides insights into the association between regional cholinergic deficits and PIGD motor dysfunction in PSP, offering potential avenues for improving diagnosis, prognostication, and treatment of these challenging neurodegenerative disorders. The observed widespread reductions of regional cholinergic terminal density across various brain regions emphasizes the complex interplay between neurotransmitter systems and clinical manifestations of the disease. Further research is warranted to elucidate the specific mechanisms underlying these cholinergic deficits in PSP.

In conclusion, our study suggests that PIGD symptoms in PSP are associated with cholinergic degeneration across an assemblage of brain regions, indicating the involvement of multifactorial deficits, including cognitive, multi-sensory, oculomotor, and emotional components. These regions significantly overlap with those found to be associated with PIGD motor features in PD, suggesting that the cholinergic system may have inherent vulnerabilities to neurodegeneration, independent of specific proteinopathies. Our study suggests that the degeneration of cholinergic innervation in thalamic, striatal, limbic, and frontal brain structures may underlie the debilitating symptoms in PSP and could serve as a target for future interventions. A novel finding is that cholinergic forebrain losses are also associated with PIGD motor features in PSP. Future cholinergic enhancement strategies, such as subtype specific cholinergic receptor agents, may offer avenues for mitigating motor dysfunction in individuals with PSP.

## Supplementary Material

Supplementary Files

This is a list of supplementary files associated with this preprint. Click to download.

- SupplementaryMaterials.docx

## Figures and Tables

**Table 1:** Clinical descriptions of study subjects based on the Movement Disorder Society clinical diagnosis criteria for PSP (MDS-PSP).

Patient number	Sites	Ocular Motor Dysfunction	Postural Instability	Akinesia	Cognitive Dysfunction	PSP Rating Scale Total	Subtype
PSP01	Michigan	O1	P1	--	--	--	Probable PSP-RS
PSP02	Michigan	O2	P1	A3	C1	--	Probable PSP-RS
PSP03	Michigan	O1	P1	--	C1	--	Probable PSP-RS
PSP04	Michigan	O1	unavailable	A2	--	--	Probable PSP*
PSP05	Michigan	O2	P1	A2	--	--	Probable PSP-RS
PSP06	Michigan	O1	unavailable	A2	--	--	Probable PSP*
PSP07	Michigan	O1	P2	A1	--	34	Probable PSP-RS
PSP08	Michigan	O1	P1	--	C3	53	Probable PSP-RS
PSP09	Michigan	O1	P1	A1	--	30	Probable PSP-RS
PSP10	Michigan	O1	P1	A1	--	24	Probable PSP-RS
PSP11	Michigan	O1	--	A2	--	24	Probable PSP-P
PSP12	Michigan	O1	P1	A1	C2	47	Probable PSP-RS
PSP13	Michigan	O1	P1	--	C2	45	Probable PSP-RS
PSP14	Michigan	O2	P1	A1	--	25	Probable PSP-RS
PSP15	Michigan	O1	P1	A2	C2	--	Probable PSP-RS
PSP16	Michigan	O2	P1	A3	-	35	Probable PSP-RS
PSP17	Groningen	--	--	A3	C2	--	Suggestive PSP-P
PSP18	Groningen	--	P1	A2	--	--	Suggestive PSP-P
PSP19	Groningen	--	--	A3	C2	--	Suggestive PSP-P

* Denotes those patients in whom details regarding the timing of postural instability relative to their disease onset was unavailable. In those cases, formal subtyping was not pursued to avoid misclassification.

**Table 2:** Demographic, clinical, motor and cognitive details for PSP Participants

Demographics	Mean (SD)
Age	69.47 (6.46)
Gender	Females: 7 Males: 12
Disease Duration (years from first symptom)	5.2 (3.70)
LED (mg)	255.26 (304.09)
**Motor Assessments**	
Hoehn & Yahr stage	3.36 (1.22)
MDS-UPDRS Part III	42.37 (13.52)
**Neuropsychological Assessments**	
MoCA	22.58 (5.49)

**Table 3:** Significant PIGD-associated reduced [^18^F]FEOBV binding clusters (minimum 50 voxels) using SPM voxel-based morphometry analysis corrected for multiple comparisons using cluster-level method corrected at FDR (p < 0.001, q < 0.05) showing the peak voxel location, t-values, and associated brain regions for each cluster.

Cluster (voxels)	Peak MNI Coordinates			BA	Peak t-value	Peak Voxel location	Predominant Network Hub
	X	Y	Z				
6080	32	10	−30	13,18 19,20 21,22 27,28 30,34 35,36 37,38 44,45 47	8.053	Right superior temporal pole	Right Amygdala Right Calcarine Right lobule IV, V, VI, VIIb, VIII of cerebellar hemisphere Right Crus I and II of cerebellar hemisphere Right Posterior cingulate gyrus Right Inferior frontal gyrus Right Fusiform Right Heschl Right Hippocampus Right Insula Right Lingual Right Inferior occipital gyrus Right Posterior orbital gyrus Right ParaHippocampal gyrus Right Precuneus Right Rolandic operculum Right Inferior temporal gyrus Right Middle temporal gyrus Right Superior temporal pole Right Lateral geniculate nuclei Right Pulvinar
3567	−14	−42	4	18,19 20,21 23,27 28,29 30,34 35	8.579	Left Precuneus	Left Calcarine Left lobule IV, V, VI, VIIb, VIII of cerebellar hemisphere Left Crus I and II of cerebellar hemisphere Left Posterior cingulate gyrus Left Fusiform Left Hippocampus Left Lingual Left Inferior occipital gyrus Left ParaHippocampal gyrus Left Precuneus Left Inferior temporal gyrus Left Middle temporal gyrus Left Superior temporal gyrus Left Lateral geniculate nuclei Left Pulvinar Lobule IV, V and V of vermis
3022	14	32	−24	9,10 11,24 25,32 47	8.14	Right medial orbital frontal cortex	Left Anterior cingulate cortex Right Anterior cingulate cortex Left Caudate Right Caudate Right Inferior frontal gyrus Right Middle frontal gyrus Left Superior frontal gyrus Right Superior frontal gyrus Left Nucleus accumbens Right Nucleus accumbens Right Anterior orbital gyrus Right Lateral orbital gyrus Left Medial orbital gyrus Right Medial orbital gyrus Left Olfactory cortex Right Olfactory cortex Left Putamen Left Rectus Right Rectus
1553	−54	−24	6	6,13 21,22 38,40 41,42 43,45 47	5.85	Left superior temporal gyrus	Left Inferior frontal gyrus Left Heschl Left Insula Left Postcentral Left Rolandic operculum Left supramarginal gyrus Left Superior temporal gyrus Left superior temporal gyrus
938	−8	−6	6		5.89	Left VA thalamus	Right Caudate Left Anterior nucleus Right Anterior nucleus Left Intralaminar nucleus Right Intralaminar nucleus Left Lateral posterior nucleus Right Lateral posterior nucleus Left Mediodorsal nucleus Right Mediodorsal nucleus Left Pulvinar Right Pulvinar Left Ventral anterior nucleus Right Ventral anterior nucleus Left Ventral lateral nucleus Right Ventral lateral nucleus Left Ventral posterolateral Right Ventral posterolateral
560	50	−20	12	13,22 40,41 42,43	5.26	Right opercular Rolandic cortex	Right Heschl Right Insula Right Rolandic operculum Right Supramarginal gyrus Right superior temporal gyrus
282	−2	−38	24	23,31	6.58	Left posterior cingulate	Left Middle cingulate gyrus Right Middle cingulate gyrus Left Posterior cingulate gyrus Right Posterior cingulate gyrus
278	16	18	36	6,9 24,32	6.43	Right middle cingulate	Right anterior cingulate cortex Right Middle cingulate gyrus Right Superior frontal gyrus Right Supplementary motor area

## Data Availability

The data that support the findings of this study are available from the corresponding author, upon reasonable request and legal status compliance of international data.
